# Investigation of Biomarkers in Allergic Patients with Long COVID

**DOI:** 10.3390/jpm16010031

**Published:** 2026-01-05

**Authors:** Fabio Romano Selvi, David Longhino, Gabriele Lucca, Ilaria Baglivo, Maria Antonietta Zavarella, Chiara Laface, Laura Bruno, Sara Gamberale, Ludovica Fabbroni, Angela Rizzi, Arianna Aruanno, Rosa Buonagura, Marina Curci, Alessandro Buonomo, Marinella Viola, Gianluca Ianiro, Francesco Landi, Matteo Tosato, Antonio Gasbarrini, Cristiano Caruso

**Affiliations:** 1 UOSD Allergy and Clinical Immunology Unit, Fondazione Policlinico Universitario A. Gemelli, Istituto di Ricovero e Cura a Carattere Scientifico, Università Cattolica del Sacro Cuore, 00168 Rome, Italy; fabioselvi1998@gmail.com (F.R.S.); david.longhino@gmail.com (D.L.); gabbo.lucca@gmail.com (G.L.); ilaria.baglivo@guest.policlinicogemelli.it (I.B.); mariaantonietta.zavarella@policlinicogemelli.it (M.A.Z.); chiara.laface01@gmail.com (C.L.); laurabruno1097@gmail.com (L.B.); gamberalesara@gmail.com (S.G.); ludovicafabbroni@gmail.com (L.F.); angela.rizzi@policlinicogemelli.it (A.R.); arianna.aruanno@policlinicogemelli.it (A.A.); alessandro.buonomo@unicatt.it (A.B.); marinella.viola@policlinicogemelli.it (M.V.); 2 Division of Internal Medicine and Clinical Immunology, Department of Internal Medicine and Clinical Complexity, University of Naples Federico II, 80138 Naples, Italy; buonagura.rosa@gmail.com; 3 Department of Medical and Surgical Science, University of Foggia, 71122 Foggia, Italy; marinacurci2@gmail.com; 4 Centre for Digestive Disease (CEMAD), Gastroenterology Unit, Fondazione Policlinico Universitario A. Gemelli, Istituto di Ricovero e Cura a Carattere Scientifico, 00118 Rome, Italy; gianluca.ianiro@unicatt.it (G.I.); antonio.gasbarrini@unicatt.it (A.G.); 5 Department of Geriatrics, Orthopedics and Rheumatology, Fondazione Policlinico Universitario A. Gemelli, Istituto di Ricovero e Cura a Carattere Scientifico, Università Cattolica del Sacro Cuore, 00168 Rome, Italy; francesco.landi@unicatt.it (F.L.); matteo.tosato@unicatt.it (M.T.)

**Keywords:** long COVID, allergy, eosinophil cationic protein (ECP), immunoglobulin E (IgE), free light chains (FLCs), biomarkers, epithelial barrier dysfunction, immune dysregulation

## Abstract

**Background**: Long COVID remains a challenging and heterogeneous condition, with mechanisms that are still incompletely understood. Emerging evidence suggests that patients with allergic disease may experience more persistent post-COVID symptoms, possibly due to immune dysregulation and epithelial barrier fragility. **Methods**: We carried out an observational, single-center study at the Allergy and Clinical Immunology Unit of Policlinico Universitario A. Gemelli IRCCS (Rome, Italy). Seventeen adults with confirmed allergic disease and long COVID were evaluated between July and December 2024. Biomarkers reflecting allergic inflammation and barrier integrity, blood eosinophil count, total immunoglobulin E (IgE), eosinophil cationic protein (ECP), and serum free light chains (FLCs), were measured and analyzed for interrelationships and symptom correlations. **Results**: Participants (10 men, 7 women; mean age 43.7 years) showed variable biomarker profiles, consistent with the heterogeneity of allergic inflammation. Mean eosinophil count was 179 ± 72 cells/µL, total IgE 165.4 ± 140.6 kU/L, ECP 64.2 ± 48.5 ng/mL, and the kappa/lambda FLC ratio 1.20 ± 0.69. Notably, elevated kappa FLC levels (>19.4 mg/L) were significantly associated with high ECP (>20 ng/mL) (χ^2^ = 10.6, *p* = 0.001) and increased IgE (>200 kU/L) (χ^2^ = 6.0, *p* = 0.015). Individuals with higher ECP and FLCs more often reported respiratory and systemic symptoms, especially fatigue, dyspnea, and cognitive fog, that persisted beyond six months. **Conclusions**: These findings suggest that biomarkers of allergic inflammation and barrier dysfunction, particularly ECP and FLCs, may contribute to the persistence of long-COVID symptoms in allergic patients. The observed links between humoral activation, eosinophilic activity, and prolonged symptom burden support a model of sustained inflammation and delayed epithelial recovery. Larger, longitudinal studies including non-allergic controls are warranted to confirm these associations and to explore whether restoring barrier integrity could shorten recovery trajectories in this vulnerable population.

## 1. Introduction

More than four years after the start of the COVID-19 pandemic, a growing number of individuals have reported persistent symptoms extending well beyond the acute phase of infection [1,2]. Many people who once had a “mild” case of COVID-19 continue to deal with symptoms that last for months, sometimes years, after viral clearance. This condition, known as long COVID or post-COVID-19 syndrome, is recognized by the World Health Organization (WHO) as symptoms lasting at least three months after the initial infection and persisting for at least two months without another identifiable cause [1].

The list of symptoms is long, but certain ones recur: profound fatigue, shortness of breath, cognitive impairment (“brain fog”), chest tightness, and sleep disturbance. For many, these symptoms fluctuate over time, disrupting work, relationships, and overall quality of life [3,4].

Despite growing awareness, the biological mechanisms of long COVID remain incompletely understood. Several pathways have been proposed, including viral persistence, immune dysregulation, endothelial injury, and epithelial barrier dysfunction [5,6,7,8,9]. Notably, people with allergic conditions, such as asthma, allergic rhinitis, or atopic dermatitis, appear to report lingering post-COVID symptoms more frequently than others [10,11,12].

Allergic diseases share two core features: immune hyper-reactivity and epithelial barrier dysfunction [3,4]. In such individuals, skin or mucosal surfaces show increased permeability and chronic inflammation, which may predispose them to sustained post-viral inflammation [3,9]. Similar barrier abnormalities have been observed in long-COVID patients, particularly within the airway and olfactory mucosa [8,12].

This overlap suggests a shared vulnerability: the same biological mechanisms that drive allergic inflammation may also hinder recovery from SARS-CoV-2 infection [13]. Recent evidence indicates that allergic or type-2–driven airway disease involves altered expression of viral entry factors such as ACE2 and TMPRSS2, potentially influencing infection outcomes and recovery [6,14,15].

As previously reported by Caruso and colleagues at Policlinico Universitario A. Gemelli IRCCS, they previously examined asthma management and immune modulation during and after SARS-CoV-2 infection [16,17,18]. Their work supports the hypothesis that allergic individuals may exhibit persistent immune activation even after viral clearance.

This study was designed to explore that question. We evaluated biomarkers reflecting allergic inflammation and barrier integrity, blood eosinophil count, immunoglobulin E (IgE), eosinophil cationic protein (ECP), and serum free light chains (FLCs) [19,20,21,22,23]. By examining their relationships and comparing them with patients’ symptom profiles, we aimed to clarify why allergic individuals may be more prone to prolonged post-viral inflammation and to consider implications for management [24]. Several groups have reported mast cell activation–like features in long COVID, with increased prevalence of mast cell activation symptoms and, in some cohorts, elevated tryptase or MMP-9 [25,26,27]. In spite of this, we focused on blood eosinophil count, total IgE, ECP, and serum FLCs because these markers are routinely available in our clinical practice, directly reflect type-2 allergic inflammation and epithelial barrier injury, and can be repeatedly measured in outpatient long-COVID follow-up. In contrast, other potentially relevant mediators such as plasma histamine, tryptase, chymase, matrix metalloproteinase-9 (MMP-9), osteopontin, or IL-6 often require specialized assays, are less standardized in our clinical laboratory, and were therefore beyond the scope of this exploratory single-center study.

## 2. Materials and Methods

### 2.1. Study Design

We conducted an observational, single-center study at the Allergy and Clinical Immunology Unit of Policlinico Universitario A. Gemelli IRCCS (Rome, Italy) between July and December 2024. The study focused on patients with established allergic disease who continued to experience symptoms long after confirmed SARS-CoV-2 infection [9,11,13]. Given the exploratory nature of this pilot study, we aimed to describe our cohort and procedures as clearly as possible. All participants were seen consecutively in our dedicated post-COVID clinic within the Allergy and Clinical Immunology Unit, which ensured a consistent clinical setting for follow-up. During each visit, clinicians conducted structured interviews that focused on respiratory, systemic, and neurocognitive symptoms, and recorded the timing of these complaints in relation to the initial SARS-CoV-2 infection.

### 2.2. Participants

Seventeen adults (10 men, 7 women) were enrolled. Inclusion criteria were:

- laboratory confirmed COVID-19 (PCR),
- symptoms persisting ≥12 weeks after acute infection,
- a clinician-diagnosed allergic disease (asthma, allergic rhinitis, or atopic dermatitis) [3,4,28].

Specific sensitizations were known from prior allergy assessments, but specific IgE was not re-measured, as our aim was to evaluate systemic biomarkers of immune activation rather than sensitization patterns.

Written informed consent was obtained from all subjects involved in the study. A blank copy of the consent form has been provided to the Editorial Office for record keeping and is not publicly available [1,2]. The study was conducted in accordance with the Declaration of Helsinki and approved by the Ethics Committee of the Fondazione Policlinico Universitario A. Gemelli IRCCS, Rome, Italy. Ethical approval was granted as part of the institutional research program on allergic diseases and post-COVID conditions, and the reference details are available from the corresponding author upon reasonable request.

### 2.3. Data Collection

We recorded demographics (age, sex, BMI), allergic diagnoses, and symptom duration. Blood samples obtained between 90 and 240 days after initial SARS-CoV-2 symptom onset during routine visits were analyzed for:

- Eosinophil count (cells/µL), marker of allergic inflammation and tissue involvement [18,22,23].
- Total immunoglobulin E (IgE; kU/L), indicator of atopic sensitization [19,20,22].
- Eosinophil cationic protein (ECP; ng/mL), marker of eosinophil activation and tissue injury [18,19,23].
- Free light chains (FLCs) (kappa and lambda (mg/L)), the kappa/lambda ratio was used to assess immunoglobulin balance and B-cell activation [7,20,21].

All biomarkers were chosen based on previous reports linking eosinophilic and humoral activity to COVID-19 outcomes and allergic inflammation [7,20,29,30,31,32].

The selected biomarkers were chosen to capture eosinophil-driven inflammation, IgE-mediated atopy, and B-cell activation/barrier dysfunction with tests that are routinely used in our allergy clinic.

Pre-COVID biomarker measurements (eosinophils, total IgE, ECP, and FLCs) were not consistently available in our electronic records, as many patients had been followed externally prior to referral; therefore, only post-COVID samples obtained during standardized follow-up visits were analyzed.

Medication history was also reviewed to contextualize biomarker interpretation. No participants were receiving systemic corticosteroids, biologics, or immunosuppressants at the time of sampling. Eight individuals were taking oral H1-antihistamines for allergic rhinitis; these medications are not known to significantly alter ECP or free light chain concentrations.

Symptom persistence was assessed through a structured clinical interview following our unit’s standard long-COVID follow-up protocol. Symptom clusters were assigned a priori based on standard clinical categories (respiratory, systemic, and neurocognitive symptoms) rather than generated statistically, as the aim was to provide a descriptive framework for comparing biomarker patterns across common long-COVID domains.

### 2.4. Statistical Analysis

Descriptive statistics were calculated for all variables. Continuous variables are reported as mean ± standard deviation (SD) and range; categorical data as counts and percentages. Normality was assessed with the Kolmogorov–Smirnov test. Associations between dichotomized biomarkers were tested with Pearson’s chi-square (χ^2^) on 2 × 2 tables (df = 1).

Cut-off points were selected *a priori* using local laboratory reference limits (kappa FLC > 19.4 mg/L; lambda FLC > 26.3 mg/L; ECP > 20 ng/mL) and a clinically meaningful IgE threshold (>200 kU/L) commonly used to denote markedly elevated atopy or biologic candidacy [19,20,23].

Statistical significance was set at *p* < 0.05. Analyses were performed with SPSS v25.

**Figure 1:**
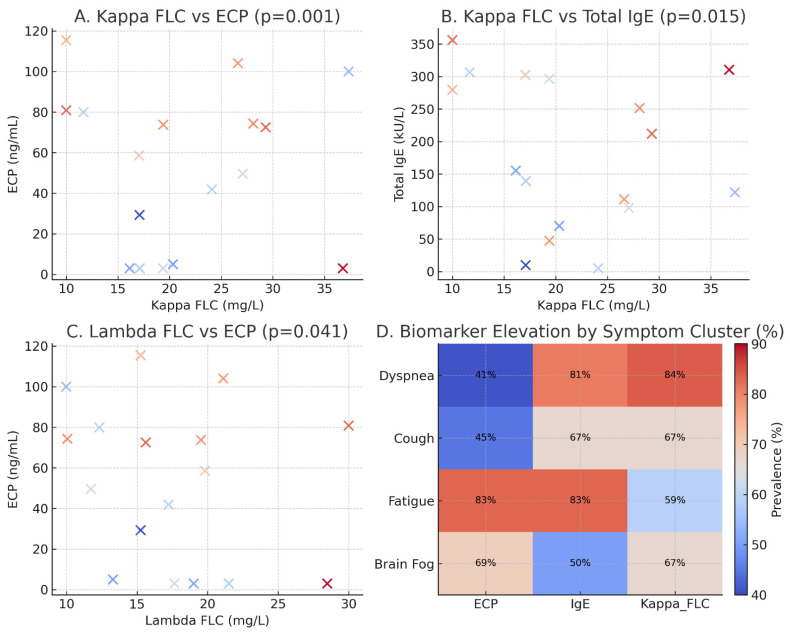
Associations between dichotomized biomarker categories, with *p*-values derived from Pearson’s chi-square tests. (**A**) Kappa free light chains (FLC) strongly correlated with eosinophil cationic protein (ECP) (*p* = 0.001). (**B**) Elevated Kappa FLCs were also associated with higher total IgE (*p* = 0.015). (**C**) Lambda FLC correlated modestly with ECP (*p* = 0.041). (**D**) Heatmap showing the proportion of patients with biomarker elevations across major symptom clusters (dyspnea, cough, fatigue, and brain fog). Data highlight coordinated activation of humoral and eosinophilic pathways in allergic individuals with long COVID, supporting a model of sustained inflammation and epithelial barrier dysfunction.

Distribution patterns were evaluated to determine whether continuous variables could be analyzed meaningfully in their original scale. Given the small sample size, biomarker comparisons were performed using dichotomized variables rather than continuous statistics to reduce the influence of outliers. Pearson’s chi-square test with continuity correction was applied for categorical associations; Fisher’s exact test was avoided because several 2 × 2 tables contained zero-frequency cells, making exact estimation unstable. As all analyses were based on categorical thresholds, correlation coefficients (r, ρ, τ) were not calculated. The *p*-values reported in Figure 1A–C were derived from Pearson’s chi-square tests applied to dichotomized biomarker categories, consistent with the categorical analytic approach described above. Formal group comparisons using parametric or non-parametric tests (*t*-test or Mann–Whitney) were not performed because the sample size was insufficient for reliable continuous-variable inference and distributions were markedly skewed.

## 3. Results

### 3.1. Participant Characteristics

Seventeen allergic long COVID patients were included (10 men, 7 women); the mean age was 43.7 years (range 25–64). All reported symptoms persisting >3 months after infection, consistent with the WHO definition of post-COVID-19 condition [1]. Allergic diagnoses included asthma, allergic rhinitis, and atopic dermatitis, with overlapping conditions in several participants [3,4,28]. To provide a more comprehensive characterization of the cohort, we expanded the descriptive analysis of symptomatic domains. Fatigue (76%), dyspnea (58%), cough (41%), and cognitive difficulties (47%) were the most frequently reported symptoms. Symptom duration ranged from 3 to 18 months, with a median persistence of 7.5 months.

### 3.2. Biomarker Profiles

Biomarker levels showed substantial interindividual variability, consistent with prior studies on allergic inflammation and immune heterogeneity [18,19,20,21,22,23].

1. Total IgE levels demonstrated a wide range (5–844 kU/L), with a mean of 165.4 kU/L (SD ± 140.6), reflecting the individualized nature of allergic sensitization among participants [22,23]. It should also be noted that elevated total IgE may reflect broader type-2 inflammatory activity rather than sensitization alone, and therefore may capture immune dysregulation extending beyond classical atopic traits.
2. Kappa FLC: mean 21.7 ± 9.9 mg/L
3. Lambda FLC: mean 19.1 ± 6.4 mg/L
4. The calculated Kappa/Lambda ratio varied from 0.6 to 3.5 (mean: 1.20, SD ± 0.69), suggesting some degree of immune imbalance across the cohort [7,20,21].
5. ECP levels showed marked fluctuation with concentrations ranging from 3 to 254 ng/mL. The mean value was 64.2 ng/mL (SD ± 48.5), consistent with heightened eosinophilic activity in many subjects [18,19,23].

All characteristics and biomarkers are shown below in Table 1.

We additionally examined the distribution of biomarker values using density plots and IQR ranges, which confirmed the presence of right-skewed distributions for IgE and ECP, consistent with the known heterogeneity of allergic inflammation. FLCs showed a narrower distribution but demonstrated meaningful clustering above the upper reference limit in several subjects.

### 3.3. Correlations Between Biomarkers

A notable co-occurrence was observed between free light chains (FLCs),particularly Kappa and Lambda subtypes, and elevated levels of Eosinophil Cationic Protein (ECP) and total IgE, in line with other observations linking these markers to airway inflammation [18,20,21,23].

A Pearson’s Chi-square test demonstrated a significant relationship between elevated Kappa FLCs (>19.4 mg/L) and high ECP levels (>20 ng/mL). Specifically, 6 individuals had both Kappa levels above 19.4 mg/L and ECP levels above 20 ng/mL, while no individuals with Kappa levels below 19.4 mg/L had elevated ECP levels. This association was statistically significant (χ^2^ = 10.6, *p* = 0.001), suggesting a strong link between increased Kappa light chain activity and eosinophilic activation [21].

Similarly, elevated total IgE levels (>200 kU/L) were observed in 4 individuals who also had Kappa levels above 19.4 mg/L, while no such elevation was noted among participants with lower Kappa levels. This relationship was also statistically significant (χ^2^ = 6.0, *p* = 0.015), indicating a potential immunologic correlation between IgE production and Kappa light chain elevation [21,22]. Because this correlation involved only four patients, the result should be considered observational and descriptive; no causal inferences can be drawn, and the statistical signal may reflect small-sample variability rather than a reproducible association.

A smaller, yet statistically significant association was seen between elevated lambda FLC (>26.3 mg/L) and ECP > 20 ng/mL (χ^2^(1, n = 17) = 4.1, *p* = 0.041), warranting further exploration despite the limited number of cases [7,20,21]. These relationships are illustrated in Figure 1.

### 3.4. Clinical Correlations

Statistical analysis revealed several meaningful correlations:

Patients with elevated eosinophil counts and total IgE levels were more likely to report prominent respiratory symptoms, including persistent dyspnea and chronic cough [9,10,12,28]. Elevated levels of both ECP and FLCs were more frequently associated with broader systemic symptomatology, particularly increased frequency of upper respiratory infections and worsening of allergic baseline symptoms [18,23,33].

**Figure 2:**
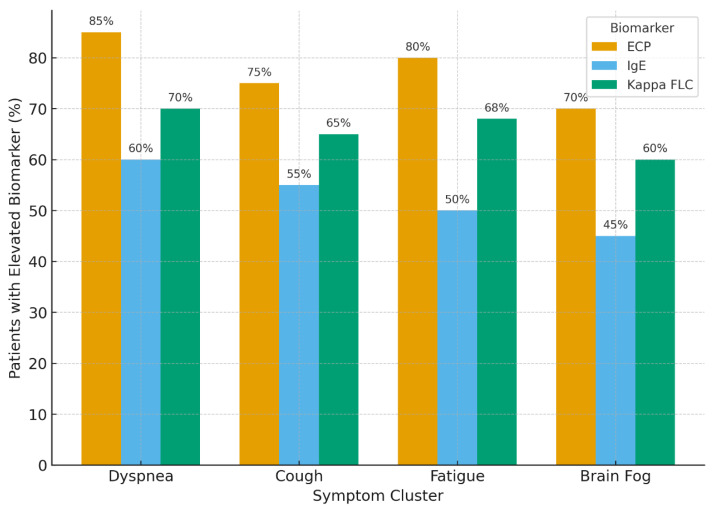
Proportion of allergic long-COVID patients with elevated biomarkers by symptom cluster. Bars represent the percentage of patients exhibiting elevated eosinophil cationic protein (ECP > 20 ng/mL), total IgE (>200 kU/L), or Kappa free light chains (>19.4 mg/L) among those reporting persistent dyspnea, cough, fatigue, or brain fog. Elevated ECP and Kappa FLC were most frequent in patients with respiratory symptoms, whereas systemic symptoms such as fatigue and cognitive disturbance showed moderate but consistent biomarker elevation, suggesting a shared inflammatory pathway linked to epithelial barrier dysfunction.

Furthermore, individuals with higher ECP and FLC levels tended to experience longer recovery trajectories, with some reporting persistent fatigue, cognitive fog, and respiratory discomfort well beyond six months post-infection [7,8,9,11,34]. To aid interpretation, we created grouped bar visualizations illustrating the proportion of patients with elevated biomarkers within each symptom cluster; as shown in Figure 2, patients reporting respiratory or systemic symptoms tended to exhibit higher proportions of elevated biomarkers, providing a clearer visual interpretation of symptom–biomarker relationships. These patterns suggest that individuals with combined elevations of ECP and Kappa FLC tended to report broader multisystem involvement, supporting a link between humoral activation, eosinophil activity, and symptom persistence.

While causality cannot be determined from these observational findings, the consistent associations between biomarker elevations and symptom burden suggest a potential role for barrier dysfunction and allergic inflammation in the pathophysiology of long COVID in allergic individuals [3,4,9,13,28].

## 4. Discussion

Findings from this small cohort suggest that biomarkers of allergic inflammation and epithelial barrier dysfunction, eosinophil count, IgE, ECP, and FLCs, may help explain the persistence and expression of long COVID symptoms in allergic patients [3,4,7,18,19,20,21,22,23]. In allergic disease, immune responses are primed toward hypersensitivity; an acute SARS-CoV-2 infection may further disrupt this balance [6,14,15,28]. A subset of patients appears to remain in a sustained activation state, with incomplete down-regulation of inflammatory pathways [7,9,11].

The association of elevated kappa FLCs with ECP and IgE is noteworthy [20,21,22,23] although it should be interpreted with caution given the exploratory nature of this study. This pattern may be compatible with ongoing immune activation and impaired epithelial recovery, but further research is needed to clarify these relationships. Given the limited sample size, these associations should be viewed as preliminary signals rather than definitive evidence of a specific mechanism. Despite FLCs being most often discussed in autoimmune or hematologic contexts, their elevation here may reflect heightened humoral activity and loss of immune homeostasis [7,9,11]. In combination with high ECP, a marker of eosinophil-mediated tissue injury, this pattern is consistent with chronic immune activation and potential epithelial barrier compromise [3,4,18,19,23]. By providing a fuller description of our dataset and analyses, the overall picture becomes clearer. Even though this pilot study is not powered for formal hypothesis testing, the recurring pattern of elevated ECP, IgE, and Kappa FLC across different symptom groups suggests a shared thread of persistent, barrier-related inflammation in allergic patients with long COVID.

Barrier dysfunction may represent a relevant framework for interpreting these findings [3,4,7,8,33,34]. Epithelial surfaces in the skin, airways, and gut act as the immune system’s primary interface. In allergic individuals these barriers may exhibit increased permeability, granting access to allergens and microbes and perpetuating inflammation [13,28]. Additional disruption following SARS-CoV-2 infection could plausibly create a self-reinforcing cycle: inflammation impairs the barrier, and the impaired barrier sustains inflammation, delaying recovery [7,8,9,34].

Mast cells represent another key effector population at epithelial barriers that has been implicated in both allergic disease and long COVID [35]. Recent reports describe mast cell activation–like phenotypes, elevated tryptase or MMP-9 [36,37], and an association between mast cell mediators and long-COVID symptom burden. In this context, our observation of elevated ECP is particularly interesting, because eosinophil granule proteins can further activate or recruit mast cells, potentially amplifying a mast cell–eosinophil inflammatory loop at mucosal surfaces. While we did not directly measure mast cell mediators in this pilot cohort, our findings, although speculative, may align with emerging evidence suggesting that barrier-associated inflammatory pathways could be involved in long COVID, particularly in allergic individuals.

The apparent over-representation of allergic rhinitis among those with more persistent symptoms may reflect the vulnerability of the nasal and upper airway mucosa, which are rich in ACE2-expressing cells and prone to chronic irritation in allergic disease [6,14,15,33]. Transcriptomic evidence from recent nasal epithelial studies in long COVID also supports sustained immune activation and impaired mucociliary function [12,33].

These observations must be interpreted cautiously given the small sample size, single-center design, and the absence of a non-allergic control group [13]. Nonetheless, the internal coherence of the associations, particularly the clustering of elevated kappa FLC, ECP, and IgE, supports further study in larger, longitudinal cohorts [7,9,11,34].

### 4.1. Clinical and Research Implications

Prospectively, FLCs and ECP, alone or combined with eosinophils and IgE, could be explored as pragmatic risk stratification markers to identify allergic patients at higher risk of prolonged symptoms [7,18,20,21,22,23]. Interventional studies might evaluate whether targeted modulation of allergic inflammation (e.g., anti-IgE or anti-eosinophil biologics) shortens recovery or mitigates symptom burden in long COVID [24].

Real-world evidence from Italian and European asthma registries, including those analyzed by Caruso and colleagues at Policlinico Gemelli, emphasizes the need for precision monitoring and tailored biologic use to minimize systemic steroid exposure and long-term harm [15,24]. Integrating biomarker data into this precision framework could support earlier identification of patients likely to experience extended post-viral inflammation [9,11].

Finally, restoring epithelial barrier integrity through anti-inflammatory and mucosal-supportive strategies may represent an underappreciated therapeutic target [3,4,7,8,13,33,34]. This concept aligns with the “epithelial barrier hypothesis,” which connects allergic and autoimmune conditions through shared mechanisms of barrier fragility and dysregulated immunity [3,28].

### 4.2. Limitations

A key limitation is the absence of pre-COVID baseline biomarker data, which prevents direct comparison with patients’ pre-infection immune status; this constraint reflects the real-world outpatient setting of our cohort and further supports the exploratory nature of the study.

We did not measure mast cell-specific mediators such as histamine, tryptase, or chymase, nor other inflammatory markers such as MMP-9, osteopontin, or IL-6; this limits the completeness of our biomarker panel and should be addressed in future studies. Given the exploratory nature of the study and the potential for overinterpretation, all mechanistic considerations presented should be viewed as hypothesis-generating.

## 5. Conclusions

Our study suggests a possible link between allergic inflammation–related biomarkers and the persistence of long-COVID symptoms in allergic individuals. While these patterns are preliminary, they may warrant further investigation in larger and controlled cohorts. These exploratory findings highlight the importance of future studies aimed at clarifying whether allergic inflammation or barrier dysfunction meaningfully contribute to long-COVID trajectories. Biomarkers traditionally associated with allergic disease may hold promise not only for predicting long-COVID risk but also for guiding personalized treatment strategies [9,11,13,24,28].

Identifying high-risk patients early and addressing barrier integrity and immune balance could improve outcomes in this vulnerable subpopulation.

Future multicenter studies with longitudinal biomarker tracking and inclusion of non-allergic controls will be crucial to validate and expand upon these results.

## Figures and Tables

**Table 1:** Demographic and biomarker characteristics of study participants (n = 17).

Variable	Mean ± SD	Range
Sex (M/F)	10/7	
Age (years)	43.7 ± 10.5	25–64
BMI (kg/m^2^)	25.3 ± 2.9	20.6–30.2
Blood Eosinophils (cells/µL)	179 ± 72	40–320
Total IgE (kU/L)	165.4 ± 140.6	5–844
ECP (ng/mL)	64.2 ± 48.5	3–254
Kappa FLC (mg/L)	21.7 ± 9.9	10–45
Lambda FLC (mg/L)	19.1 ± 6.4	9–30
Kappa/Lambda ratio	1.20 ± 0.69	0.6–3.5

## Data Availability

The original contributions presented in this study are included in the article. Further inquiries can be directed to the corresponding authors.
